# Risk factors for left ventricular remodeling after myocardial infarction: A meta-analysis

**DOI:** 10.1097/MD.0000000000040496

**Published:** 2024-11-15

**Authors:** Baozhu Xu, Wenhui Li, Zhuozhi You, Nan Yang, Lanxiang Lin, Yuefeng Li

**Affiliations:** a Health Management Center, Xiang’an Hospital of Xiamen University, Xiamen, China; b Department of Cardiology, No.910 Hospital of The Chinese People’s Liberation Army Joint Logistic Support Force, Quanzhou, China.

**Keywords:** acute myocardial infarction, meta-analysis, review, risk factors, ventricular remodeling

## Abstract

**Background:**

This study aimed to assess potential risk factors for left ventricular remodeling (LVR) after acute myocardial infarction (MI).

**Methods:**

We systematically searched PubMed, the Cochrane Library, MEDLINE, Embase, Web of Science databases CNKI Scholar, VIP, and WanFang databases for all relevant epidemiological studies published up to August 1, 2023. Fixed-effects model or random-effects model was employed to pool the study-specific effect sizes and 95% confidence intervals (CIs).

**Results:**

Fifteen studies with a total of 3,093,792 participants were included according to inclusion criteria. Major modifiable risk factors associated with LVR after MI were diabetes (odds ratio [OR] = 2.053, 95% CI: 1.504–2.803), MI site (OR = 2.423, 95% CI: 1.584–3.708), cystatin C (OR = 6.204, 95% CI: 1.830–21.036), B-type natriuretic peptide (OR = 2.280, 95% CI: 1.466–3.546), as well as creatine kinase-myocardial band (OR = 1.013, 95% CI: 0.985–1.042).

**Conclusion:**

The current study provides evidence indicating that diabetes, the site of MI, cystatin C, B-type natriuretic peptide, and creatine kinase-myocardial band are the primary risk factors for LVR after MI. Recognizing and addressing these modifiable risk factors is crucial for the development of effective preventive and treatment strategies.

## 1. Introduction

Myocardial infarction (MI) is a major cause of morbidity and mortality worldwide, with more than 7 million cases annually, thus causing a great economic burden.^[1]^ Despite early and effective coronary reperfusion, acute MI frequently results in adverse left ventricular remodeling (LVR), a maladaptive process resulting from cardiac injury, characterized by morphological changes in LV structure and geometry, leading to subsequent alterations in cardiac function.^[2,3]^ LVR after MI is a complex pathophysiological condition that leads to adverse clinical outcomes, including an increased incidence of heart failure, heightened mortality rates, and a greater risk of arrhythmic events.^[4,5]^ The pathophysiology of LVR after MI is multifaceted, involving a complex interplay of neurohormonal, inflammatory, and mechanical factors. Our understanding of the risk factors that lead to adverse LVR after MI is still evolving, and there is no comprehensive synthesis of risk factors associated with LVR after MI, a limitation that impairs strategies to curb both the financial and health burden associated with LVR after MI.

Therefore, we conducted a systematic meta-analysis to synthesize existing evidence and assess the association between multiple risk factors and LVR after MI. The findings of this meta-analysis are expected to enhance our understanding of the potential risk factors for LVR after MI. This knowledge can inform clinical practice by facilitating early risk stratification and targeted interventions aimed at mitigating adverse remodeling processes. Ultimately, improving our ability to predict and manage LVR after MI holds the potential to enhance patient outcomes and reduce the burden of cardiovascular disease.

## 2. Methods

This meta-analysis was performed according to the Preferred Reporting Items for Systematic Reviews and Meta-Analyses (PRISMA) guidelines and registered in the PROSPERO database (CRD42024540683).

### 2.1. Search strategy

The search of relevant literature followed the PICO framework: (P) Patients with ventricular remodeling after acute myocardial infarction; (I) Risk factors, such as hypertension, diabetes, and coronary artery lesions; (C) Comparison with the non-outcome group; (O) Left ventricular remodeling. This systematic review and meta-analysis were performed according to the Preferred Reporting Items for Systematic Reviews and Meta-Analyses guidelines. We searched PubMed, the Cochrane Library, MEDLINE, Embase, Web of Science databases CNKI Scholar, VIP, and WanFang databases up to August 1, 2023. Combined keywords (AND/OR) were searched: remodeling (reverse and adverse); ventricular remodeling, myocardial infarction, and risk factors. The detailed search keywords are provided in the Supplementary Material, Supplemental Digital Content, http://links.lww.com/MD/N901.

### 2.2. Inclusion and exclusion criteria

Inclusion criteria: the article was published in peer-reviewed; the study aimed to test risk factors for ventricular remodeling after acute myocardial infarction; the article provided adequate data to calculate odds ratios (ORs) and 95% confidence intervals (CIs); full-text available. Exclusion criteria: data that could not be extracted; case reports, protocols, reviews, conference summaries, and dissertations; duplicate studies.

### 2.3. Data extraction and quality assessment

The screening process was conducted after excluding duplicate articles. Two reviewers independently verified the accuracy of the data, and any discrepancies or disagreements were addressed through discussion until a consensus was achieved. The quality of noncomparative studies was evaluated based on MINORS quality assessments, and a score > 17 was considered high-quality.^[6]^

### 2.4. Statistical analysis

Continuous quantitative data are presented as mean ± standard deviation (SD). If the original articles only provide median and interquartile ranges (IQR), the methods suggested by Luo et al^[7]^ and Wan et al^[8]^ were used to convert them into means and standard deviations (SDs). The categorical data was depicted by the number of cases and composition ratio n (%).

Statistical analysis was conducted using STATA 16.0 software. For survival data, hazard ratios (HRs) were combined using the generic inverse variance method.^[8]^ Heterogeneity was assessed using Cochran chi-square *Q* test, with significance set at *P* < .10 and *I*^2^ > 50%. In instances where heterogeneity was present, the random effects model was employed to estimate pooled odds ratios (ORs). Conversely, when heterogeneity was not detected, pooled ORs were estimated using the fixed-effects model.

Potential publication bias was evaluated subjectively using a funnel plot and objectively using Egger weighted correlation at a 5% significance level; a *P* < .05 indicates the existence of publication bias, and *P *≥ .05 indicates the absence of publication bias. If there was evidence of publication bias (*P* < .05), the trim and fill method was applied to adjust for the effect of publication bias.^[9]^

## 3. Results

**Figure 1.**
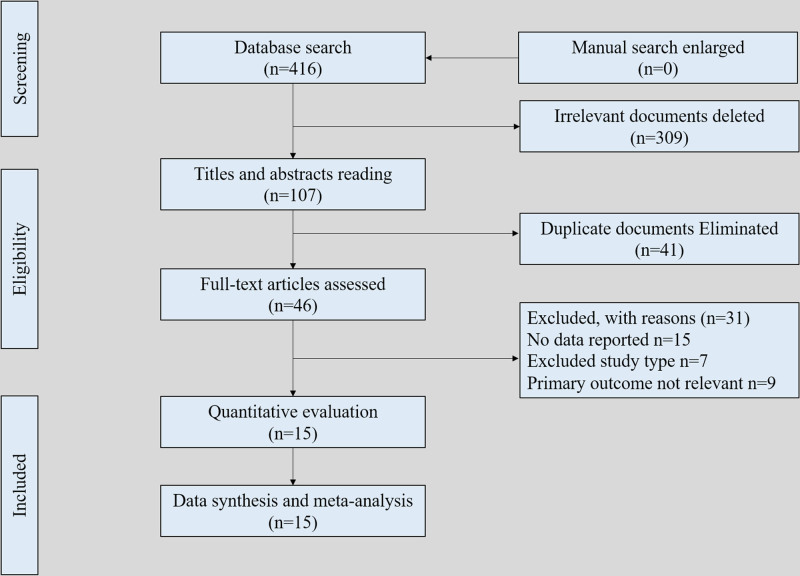
Flowchart of literature review and data extraction process.

After a systematic literature search and cross-checking of references, 416 studies were included. A total of 309 studies were removed owing to these studies reporting irrelevant topics, 41 studies were removed as duplications, then 31 studies were removed after assessing titles and abstracts. Through full-text assessments, 15 studies were finally included (Fig. 1). A total of 15 studies were identified for inclusion in the current review that reported on a total of 3093,792 patients. All of the included studies were of a high methodological quality, with the MINORS score ≥ 20 points (Table 1). The overview of potential risk factors of left ventricular remodeling after myocardial infarction is present in Table 2.

**Table 1:** Characteristics of included studies for meta-analysis.

Authors	Year	Country	Age	Sex (M/F)	Sample size	Risk factors							
						Hypertension	Diabetes	Myocardial infarction site	NT-proBNP	Coronary artery lesions	CysC	BNP	CKMB
Zhang^[10]^	2022	China	65.13 ± 12.48	60%/40%	100	NA	0.945 (0.397–2.253)	6.354 (1.446–27.924)	1.032 (0.876–1.215)	1.898 (1.655–2.176)	15.133 (5.116–44.758)	NA	NA
Wang^[11]^	2021	China	53.69 ± 12.08	79.4%/20.6%	73	NA	1.295 (0.441–3.803)	4.762 (1.733–13.082)	NA	NA	NA	NA	NA
Lin^[12]^	2021	China	64.84 ± 12.61	67.5%/32.5%	120	3.283 (0.678–9.913)	NA	NA	NA	NA	NA	1.610 (1.354–3.139)	1.312 (1.002–2.835)
Li^[13]^	2019	China	60.52 ± 5.36	54.3%/45.7%	160	9.623 (1.738–15.369)	4.325 (2.652–9.325)	NA	NA	NA	7.358 (0.365–13.265)	NA	NA
Aboelkasem^[14]^	2018	Egypt	55.66 ± 12.02	84.8%/15.2%	152	NA	NA	1.9 (1.7–2.55)	NA	2.23 (1.9–2.6)	NA	NA	NA
Mollema^[15]^	2007	Netherlands	61.2 ± 8.6	78.6%/21.4%	178	1.15 (0.53–2.51)	1.24 (0.38–4.06)	NA	NA	1.2 (0.78–1.97)	NA	NA	NA
Van der Bijl^[16]^	2020	Netherlands	60 ± 12	77%/23%	1995	NA	2.51 (1.53–4.11)	2.81 (1.78–4.42)	NA	NA	NA	NA	NA
Bauters^[17]^	2017	France	NA	NA	246	NA	NA	1.08 (1.03–1.13)	NA	0.91 (0.88–0.93)	NA	2.76 (2.00–3.80)	NA
Sandhu^[18]^	2009	Canada	60 ± 8	88%/12%	100	NA	NA	3.4 (1.0–13.9)	3.6 (1.1–12.3)	5.4 (1.4–20.3)	NA	NA	NA
Richards^[19]^	2003	New Zealand	62.4 ± 10.4	78.2%/21.8%	666	NA	1.57 (1.01–2.46)	2.13 (1.40–3.26)	NA	NA	NA	3.51 (1.08–11.50)	NA
Vergès^[20]^	2005	France	65 ± 10	71.9%28.1%	560	0.96 (0.95–0.97)	1.45 (1.22–1.72)	NA	2.22 (1.92–2.58)	NA	2.91 (2.31–3.66)	NA	NA
Yoshiyama^[21]^	2005	Japan	60.8 ± 9.3	63.4%/36.6%	94	0.360 (0.079–1.619)	5.08 (1.355–19.07)	NA	NA	NA	NA	NA	1.00 (1.000–1.001)
Bordejevic^[22]^	2021	Rome	66 ± 13	73%/27%	271	3.67 (1.38–9.73)	3.9 (1.70–9.16)	3.94 (1.70–9.16)	NA	NA	NA	NA	1.03 (1.02–1.07)
Haaf^[23]^	2011	Switzerland	64 ± 9	66%/34%	658	0.985 (0.974–0.996)	2.362 (1.351–4.130)	NA	1.014 (1.010–1.017)	NA	NA	NA	NA

BMI = body mass index, CKMB = creatine kinase MB, CysC = cystatin C, F = female , M = male, NA = not available.

**Table 2:** Risk factors of left ventricular remodeling after myocardial infarction.

Risk factors	OR	95% CI	*P*	Analysis model
Hypertension	0.980	0.938–1.023	<.05	Random effects
Diabetes	1.896	1.407–2.553	<.05	Random effects
Myocardial infarction site	2.423	1.584–3.708	<.05	Random effects
NT-proBNP	1.653	0.928–2.275	.102	Random effects
Coronary artery lesions	1.667	0.980–2.837	.060	Random effects
CysC	6.205	1.832–21.016	<.05	Random effects
BNP	2.280	1.466–3.546	<.05	Random effects
CKMB	1.013	0.985–1.042	>.05	Random effects

### 3.1. Hypertension

**Figure 2.**
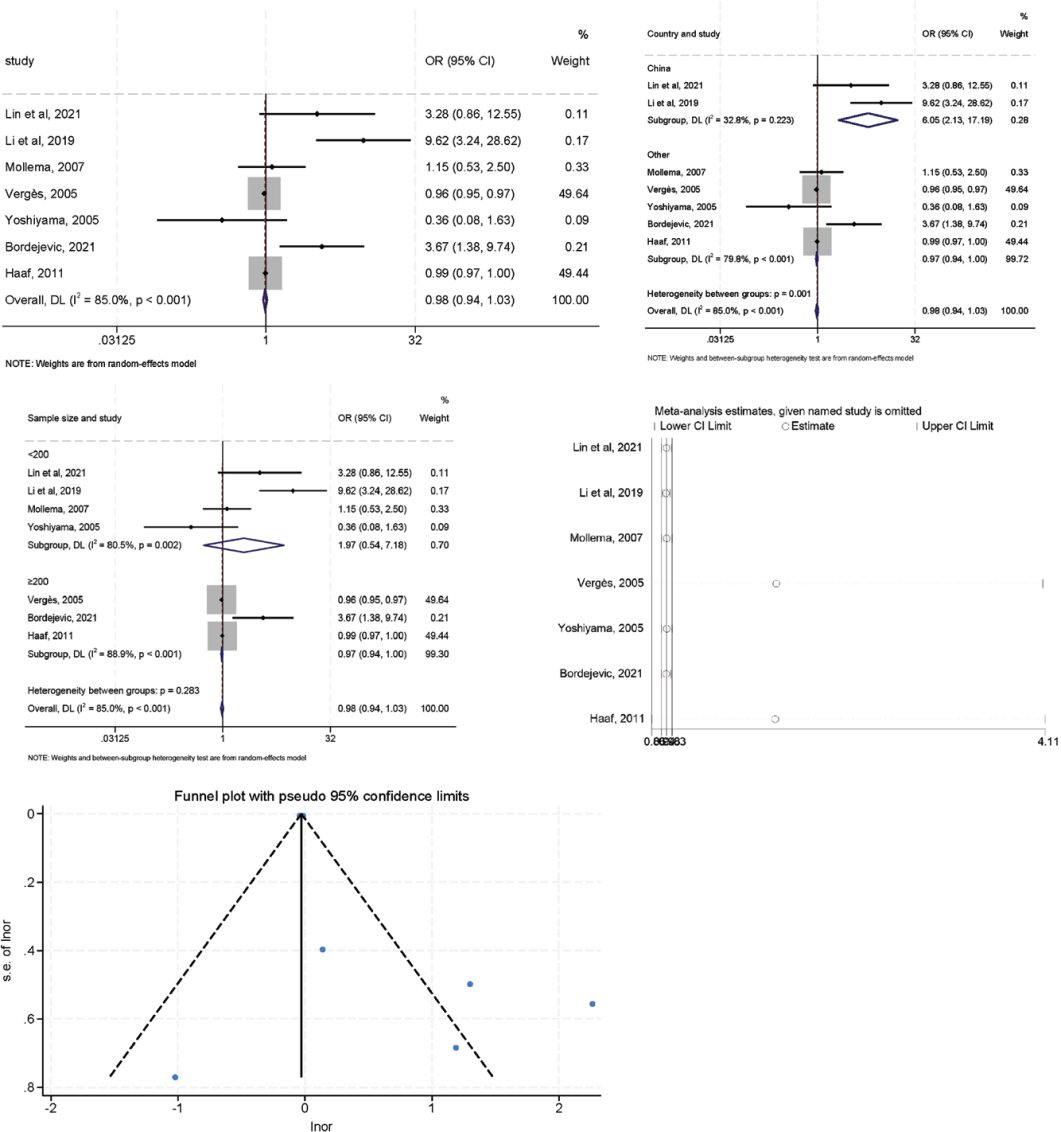
The relationship between hypertension and LVR after MI.

Due to the high heterogeneity observed in the preliminary assessment (*I*^2^ = 85.0%, *P* < .001), a random effects model analysis was performed, and the pooled OR was 0.980 (95% CI: 0.937–1.025, *P* = .377). To explore the sources of heterogeneity, further subgroup analysis was performed according to geographic region (China vs non-China) and sample size (≥200 vs <200) (Fig. 2). The heterogeneity of geographic region (China) decreased (*I*^2^ = 32.8%, *P* = .223) and the combined OR value showed a different trend (OR = 6.052, 95% CI: 2.131–17.189) suggesting that different regions of study may be 1 of the sources of heterogeneity. When the subgroup analysis was performed according to sample size, studies with cutoff values <200 showed significant association (OR = 1.974, 95% CI: 0.543–7.175), and the heterogeneity was significantly decreased when compared with the overall analysis (*I*^2^ = 80.5%, *P* = .002) meaning the sample size could be the hidden origin of heterogeneity. As a result, the high heterogeneity (*I*^2^ = 85.0%, *P* < .001) in the overall analysis might account for the subgroup analysis by geographic region and sample size. A similar trend was observed in sensitivity analyses after excluding studies from Vergès et al and Haaf et al,^[20,23]^ the OR value changed to 2.66 (95% CI: 1.18–6.00), suggesting that Vergès (2005) and Haaf (2011) study may be a major source of the heterogeneity. Furthermore, the results of Egger regression test indicated no publication bias for included studies (*t* = 1.50, *P* = .159), confirmed by the visual test of funnel plots, additional high-quality studies are needed to confirm our findings.

### 3.2. Diabetes

**Figure 3.**
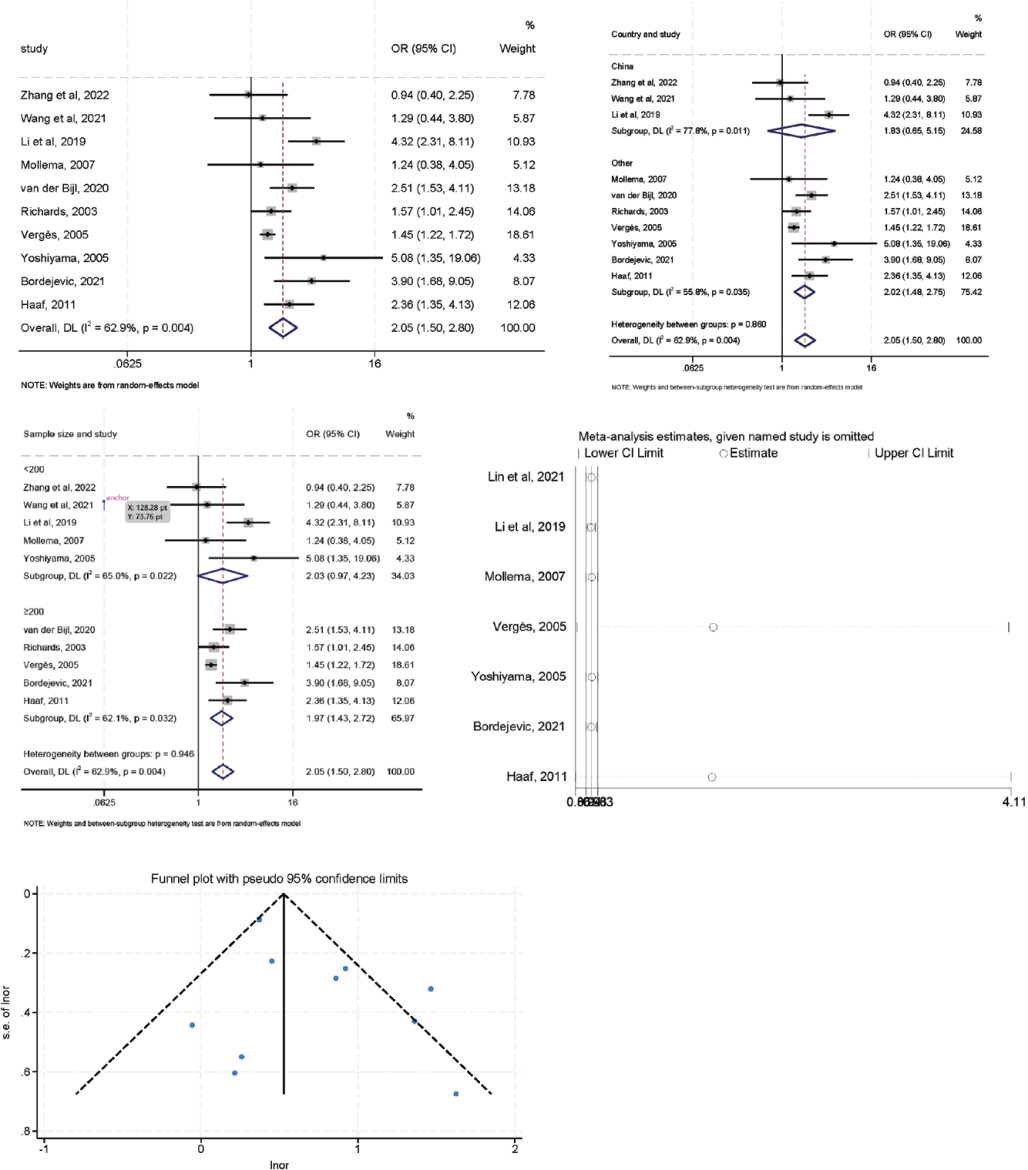
The relationship between diabetes and LVR after MI.

High heterogeneity was observed in the initial assessment (*I*^2^ = 62.9%, *P* < .05), a random effects model was used, and the pooled OR was 2.053 (95% CI: 1.504–2.803, *P* < .05). To explore the sources of heterogeneity, further subgroup analysis was performed according to geographic region (China vs non-China) and sample size (≥200 vs <200) (Fig. 3). The heterogeneity of the non-China region decreased (*I*^2^ = 48.4%, *P* = .071) and the combined OR value showed a similar trend (OR = 2.017, 95% CI: 1.478–2.752) suggesting that different regions of study maybe 1 of the sources of heterogeneity. When the subgroup analysis was performed according to sample size, both studies with cutoff values < 200 (OR = 2.029, 95% CI: 0.973–4.233) and ≥ 200 (OR = 1.974, 95% CI: 1.431–2.724) showed a significant association, and the heterogeneity was not significantly decreased. In addition, sensitivity analysis indicated that no individual study significantly influenced the combined OR, indicating that our results were reliable and robust. The Egger (*t* = 1.66, *P* = .136) tests confirmed no evidence of publication bias.

### 3.3. Myocardial infarction site

**Figure 4.**
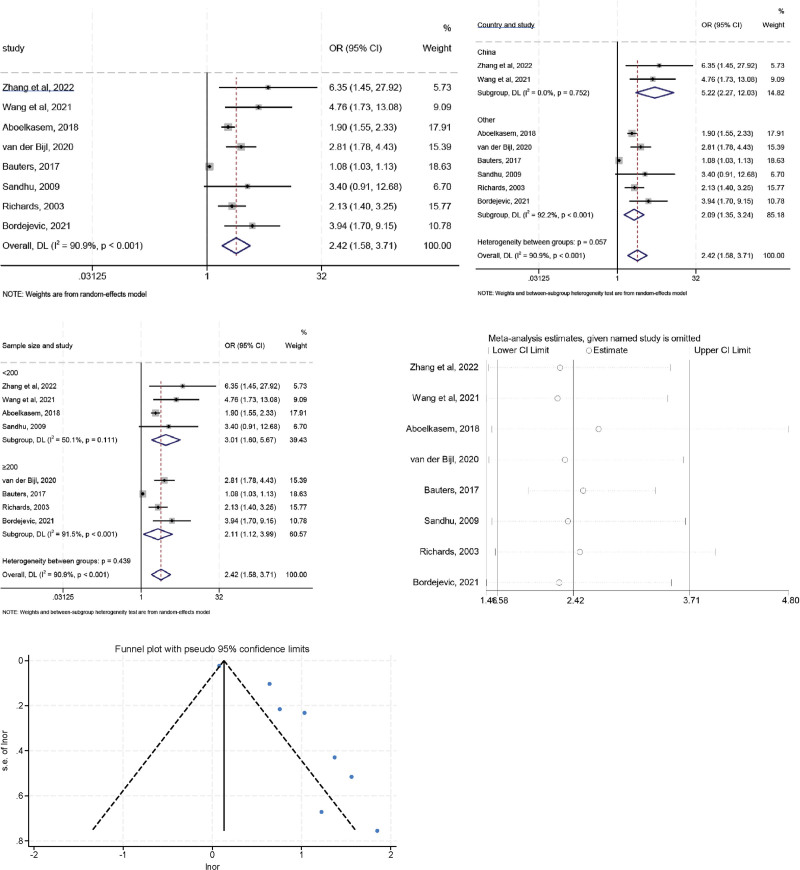
The relationship between myocardial infarction site and LVR after MI.

High heterogeneity was observed in the initial assessment (*I*^2^ = 90.9%, *P* < .001), a random effects model was used, and the pooled OR was 2.423 (95% CI: 1.584–3.708, *P* < .05). To explore the sources of heterogeneity, further subgroup analysis was performed according to geographic region (China vs non-China) and sample size (≥200 vs <200) (Fig. 4). The heterogeneity of the non-China region decreased (*I*^2^ = 0.0%, *P* = .753) and the combined OR value showed a similar trend (OR = 2.095, 95% CI: 1.354–3.242) suggesting that different regions of study maybe 1 of the sources of heterogeneity. When the subgroup analysis was performed according to sample size, studies with cutoff values < 200 showed significant association (OR = 3.008, 95% CI: 1.596–5.669), and the heterogeneity was decreased when compared with the overall analysis (*I*^2^ = 50.1%, *P* = .111) indicating that the sample size could be the hidden origin of heterogeneity. In addition, sensitivity analysis indicated that no individual study significantly influenced the combined OR, indicating that our results were reliable and robust. The Egger test indicated a significant publication bias for included studies (*t* = 5.60, *P* = .001).

### 3.4. N-terminal pro-B-type natriuretic peptide

**Figure 5.**
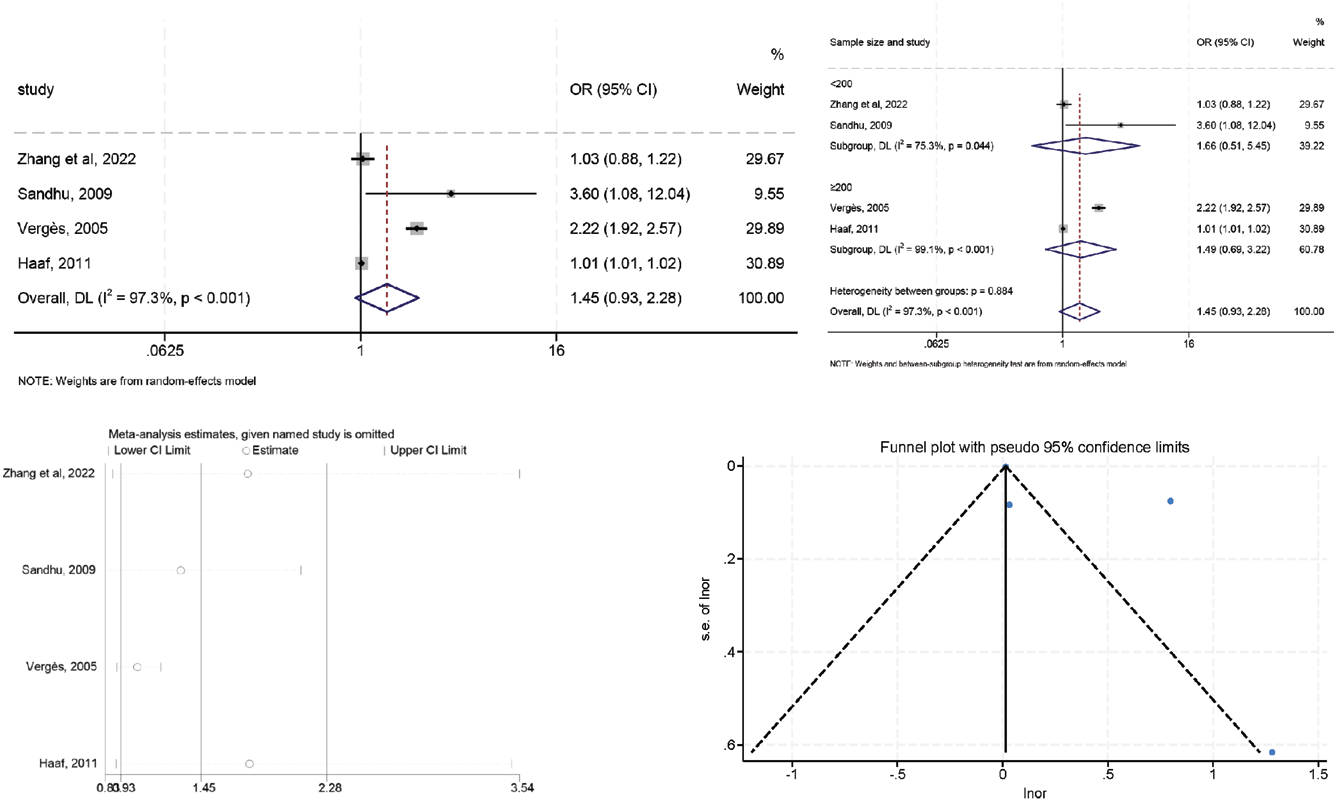
The relationship between NT-proBNP and LVR after MI.

High heterogeneity was observed in the preliminary assessment (*I*^2^ = 97.3%, *P* < .001), a random effects model was used, and the pooled OR was 1.454 (95% CI: 0.928–2.278, *P* = .102). To explore the sources of heterogeneity, further subgroup analysis was performed according to sample size (≥200 vs <200) (Fig. 5). The heterogeneity of studies with cutoff values < 200 was significantly decreased when compared with the overall analysis (*I*^2^ = 75.3%, *P* = .044) indicating that the sample size could be the origin of heterogeneity. In addition, sensitivity analysis indicated that no individual study significantly influenced the combined OR, indicating that our results were reliable and robust. The Egger (*t* = 1.34, *P* = .313) tests confirmed no evidence of publication bias.

### 3.5. Coronary artery lesions

**Figure 6.**
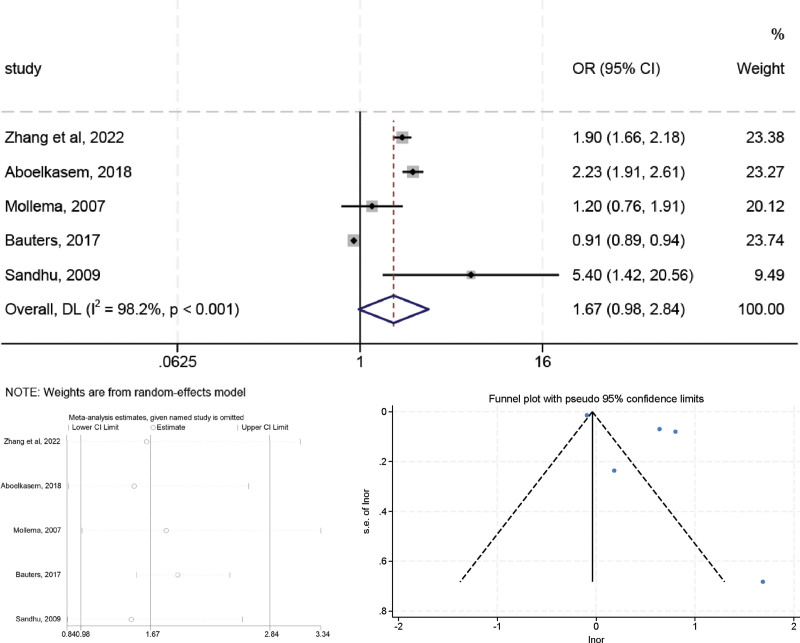
The relationship between coronary artery lesions and LVR after MI.

High heterogeneity was observed in the preliminary assessment (*I*^2^ = 98.2%, *P* < .001), a random effects model was used, and the pooled OR was 1.667 (95% CI: 0.980–2.835, *P* = .060) (Fig. 6). A similar trend was observed in sensitivity analyses after excluding studies from Bauters et al,^[17]^ the OR value changed to 1.932 (95% CI: 1.528–2.442, *P* < .05) and the heterogeneity was decreased (*I*^2^ = 67.3%, *P* = .027), suggesting that Bauters (2017) study may be a major source of the heterogeneity. The Egger (*t* = 1.99, *P* = .141) tests confirmed no evidence of publication bias.

### 3.6. Cystatin C

**Figure 7.**
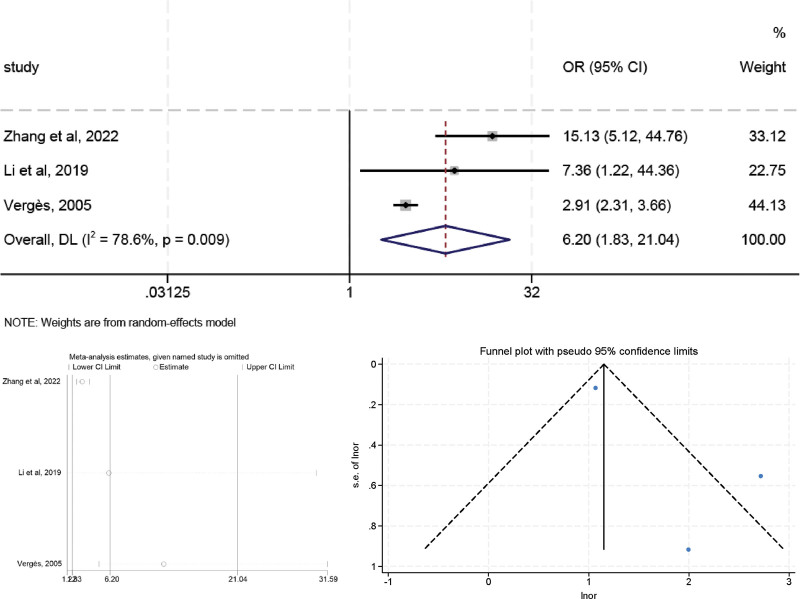
The relationship between CysC and LVR after MI.

High heterogeneity was observed in the preliminary assessment (*I*^2^ = 78.6%, *P* < .01), a random effects model was used, and the pooled OR was 6.204 (95% CI: 1.830–21.036, *P* < .05) (Fig. 7). A similar trend was observed in sensitivity analyses after excluding studies from Vergès et al,^[20]^ the OR value changed to 12.469 (95% CI: 4.933–31.517, *P* < .05) and the heterogeneity was decreased (*I*^2^ = 0.00%, *P* = .500), suggesting that Vergès (2005) study may be a major source of the heterogeneity. The Egger (*t* = 1.73, *P* = .333) tests confirmed no evidence of publication bias.

### 3.7. B-type natriuretic peptide

**Figure 8.**
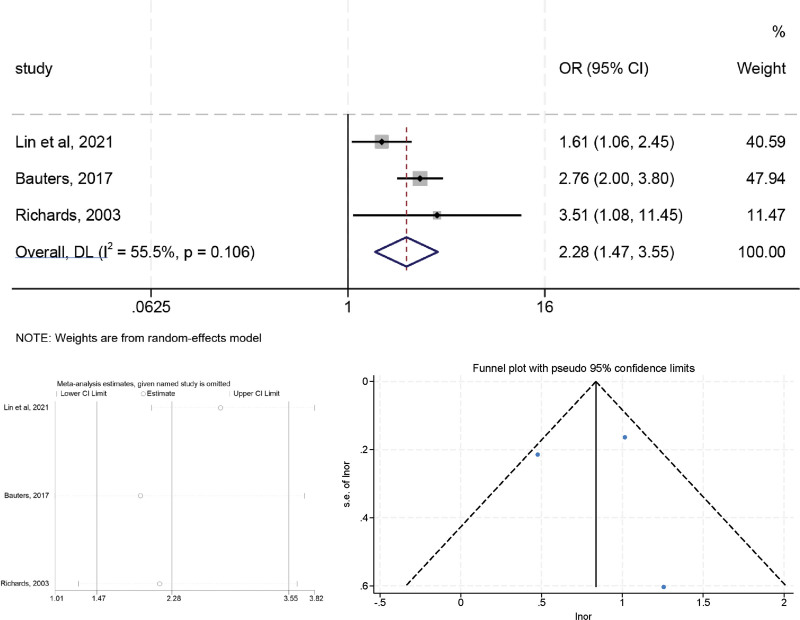
The relationship between BNP and LVR after MI.

High heterogeneity was observed in the preliminary assessment (*I*^2^ = 55.3%, *P* = .106), a random effects model was used, and the pooled OR was 2.280 (95% CI: 1.466–3.546, *P* < .05) (Fig. 8). The sensitivity analysis indicated that no individual study significantly influenced the combined OR, indicating that our results were reliable and robust. The Egger (*t* = 0.07, *P* = .956) tests confirmed no evidence of publication bias.

### 3.8. Creatine kinase-MB

**Figure 9.**
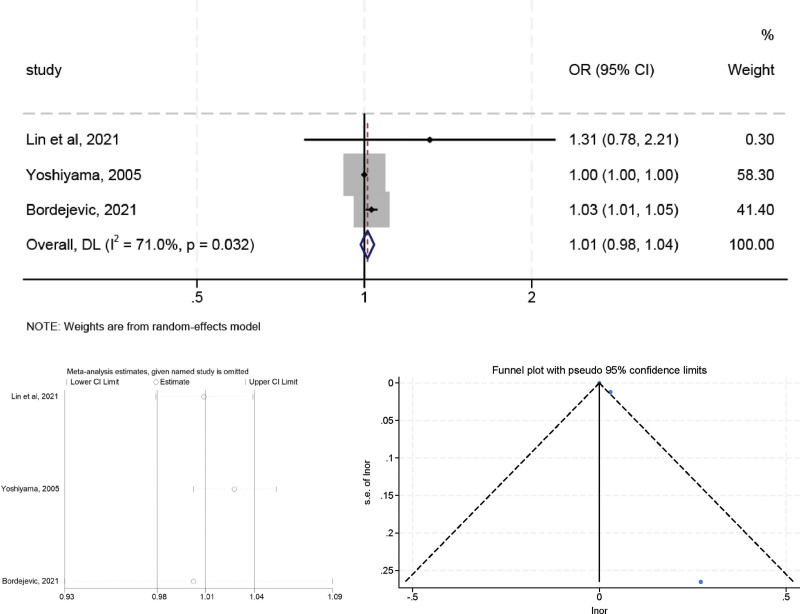
The relationship between CKMB and LVR after MI.

High heterogeneity was observed in the preliminary assessment (*I*^2^ = 71.0%, *P* < .05), a random effects model was used, and the pooled OR was 1.013 (95% CI: 0.985–1.042, *P* > .05) (Fig. 9). A similar trend was observed in sensitivity analyses after excluding studies from Yoshiyama et al,^[21]^ the OR value changed to 1.031 (95% CI: 1.006–1.055, *P* < .05) and the heterogeneity was decreased (*I*^2^ = 0.00%, *P* = .500), suggesting that Yoshiyama (2005) study may be a major source of the heterogeneity. The Egger (*t* = 2.39, *P* = .252) tests confirmed no evidence of publication bias.

### 3.9. Investigation of heterogeneity test

According to the number of included studies, we carried out the meta-regression for country and sample size to further explore the source of heterogeneity, yet none of them had shown statistical significance (*P* > .05). The detailed information is presented in Table 3.

**Table 3:** The results of meta-regressions.

Risk factors	Variable	Coefficient	Standard error	*T* value	*P* value	95% Confidence interval
Hypertension	Country	0.143	0.1350	−2.06	.108	0.010 to 1.952
	Sample size	1.079	0.642	0.13	.904	0.206 to 5.638
Diabetes	Country	1.220	0.834	0.29	.779	0.242 to 6.147
	Sample size	0.869	0.540	−0.22	.829	0.199 to 3.782
Myocardial infarction site	Country	0.412	0.277	−1.32	.245	0.073 to 2.325
	Sample size	0.929	0.441	−0.15	.884	0.274 to 3.151
NT-proBNP	Country	3.488	3.479	1.25	.429	0.0000109 to 1111952
	Sample size	0.415	0.379	−0.96	.513	3.71e−06 to 46434.47
Coronary artery lesions	Country	1.061	0.671	0.09	.933	0.069 to 16.142
	Sample size	0.451	0.283	−1.27	.333	0.030 to 6.734

## 4. Discussion

LVR after MI is a process of major importance as it leads invariably to heart failure, increased mortality, and a high economic burden. Understanding the potential risk factors that predict adverse remodeling enables a comprehensive therapeutic approach and a more accurate estimation of prognosis. In this meta-analysis of 15 studies with 3093,792 participants, modifiable risk factors associated with a higher risk of LVR after MI included hypertension, diabetes, myocardial infarction site, coronary artery lesions, cystatin C (CysC), BNP, as well as CKMB.

LVR following MI encompasses both the ischemic and remote nonischemic myocardium and occurs in 2 stages.^[24]^ The initial stage of remodeling commences at the site of the infarct within a few hours of the acute coronary occlusion and persists for approximately a week.^[25,26]^ The late phase occurs 1 month after the ischemic event and is characterized by potentially reversible structural and biochemical changes.^[27,28]^ Several risk factors, notably hypertension, and diabetes, have been identified as predictors of diastolic dysfunction in the general population,^[1,13,29]^ which is in line with our findings. Thus, early diagnosis of hypertension and effective medical intervention may reduce LVR after MI. Previous studies have indicated that non myocardial infarction (MI) patients exhibit elevated blood glucose levels, impaired glucose tolerance, and insulin resistance.^[30,31]^ Elevated level of HbA1c is independent risk factor for LVR after MI in young patients,^[32]^ suggesting early identification of diabetes or pre, coupled with timely and effective medical intervention, could help to prevent LVR after MI.

Several biomarkers of adverse cardiac remodeling have been reported in previous studies, including the heart-type fatty acid binding protein (hFABP), the ischemia-modified albumin (IMA) and the sarcomeric cardiac myosin-binding protein C (cMyC). hFABP is a low-molecular-weight, nonenzymatic protein that enters the bloodstream within an hour after the commencement of an ischemia event and returns to normal levels within 12 to 24 hours.^[33]^ Moreover, during the recovery period, hFABP levels in patients with a previous MI could predict long-term mortality and readmission due to probable heart failure.^[34]^ Sarcomeric cMyC, an isoform of myosin-binding protein found only in the heart, is a specific marker of myocardial injury that is released into the bloodstream faster than troponin, allowing earlier diagnosis of myocardial damage.^[33,35]^ Furthermore, biomarkers such as N-terminal pro-B-type natriuretic peptide (NT-proBNP), B-type natriuretic peptide (BNP), and creatine kinase-MB (CKMB) have been shown to predict adverse outcomes after MI.^[1,18,22]^ Several investigations studied the association between BNP and LV remodeling. However, the findings from these studies are inconsistent. Some relatively small studies have reported positive results,^[36,37]^ whereas a larger study reported negative results.^[38]^

Moreover, several studies have suggested that the site of myocardial infarction, with anterior infarctions potentially leading to more extensive left ventricular damage, may also influence the risk of LVR.^[10]^ Patients with anterior MI experience worse postinfarction LVR and prognosis than those with non-anterior MI, independent of the initial extent of myocardial damage.^[39,40]^ Other studies have found that patients with anterior MI have more myocardial necrosis than those with non-anterior MI, suggesting that MI size, rather than its location, is an independent predictor of postinfarction prognosis.^[41,42]^ Additionally, the number of coronary artery lesions, a measure of the extent of coronary artery disease, may also impact the risk of VR.^[19–21]^

Our study has several limitations: Some of the included studies had small sample sizes, which could impact the overall results; We focused on literature published only in English and Chinese, which may have led to selection bias by omitting studies in other languages; however, both Egger and Begg tests showed no evidence of publication bias; Most of the included studies were retrospective, raising concerns about potential selection bias that might affect the reliability of our findings; Although we detected statistical heterogeneity in our results, we were able to pinpoint some contributing factors through subgroup analyses related to hypertension, diabetes, locations of myocardial infarction, and coronary artery lesions. Still, differences in the detection methods for biomarkers such as CysC, BNP, CKMB, and NT-proBNP may significantly contribute to this heterogeneity.

## 5. Conclusion

Diabetes, the site of myocardial infarction, CysC, BNP, and CKMB are the potential risk factors for LVR following MI. Enhancing interventions targeting these modifiable risk factors is essential for reducing the incidence of heart failure and mortality.

## Acknowledgments

We are grateful to all researchers of enrolled studies.

## Author contributions

**Conceptualization:** Baozhu Xu, Yuefeng Li.

**Data curation:** Baozhu Xu, Wenhui Li, Nan Yang.

**Formal analysis:** Baozhu Xu, Wenhui Li, Nan Yang.

**Investigation:** Zhuozhi You, Lanxiang Lin, Yuefeng Li.

**Methodology:** Baozhu Xu, Wenhui Li, Zhuozhi You, Lanxiang Lin, Yuefeng Li.

**Software:** Wenhui Li.

**Supervision:** Yuefeng Li.

**Validation:** Zhuozhi You, Yuefeng Li.

**Visualization:** Zhuozhi You.

**Writing – original draft:** Baozhu Xu, Wenhui Li, Zhuozhi You, Nan Yang, Lanxiang Lin, Yuefeng Li.

**Writing – review & editing:** Baozhu Xu, Wenhui Li, Zhuozhi You, Nan Yang, Lanxiang Lin, Yuefeng Li.

## Supplementary Material
