# Supplementary material for: Risk factors for left ventricular remodeling after myocardial infarction: A meta-analysis
Source: Medicine (Baltimore). 2024 Nov 15;103(46):e40496. doi: 10.1097/MD.0000000000040496 (PMC11575972; doi:10.1097/MD.0000000000040496)
Supplement: Supplementary file 1 [file medi-103-e40496-s001.docx]

**Search Keywords**

Left Ventricular Remodeling:

"Left Ventricular Remodeling"[MeSH Terms] OR "left ventricular remodeling"[Title/Abstract] OR "cardiac remodeling"[Title/Abstract] OR "ventricular remodeling"[Title/Abstract]

Myocardial Infarction:

"Myocardial Infarction"[MeSH Terms] OR "Myocardial Infarction"[Title/Abstract] OR "Heart Attack"[Title/Abstract] OR "AMI"[Title/Abstract] OR "Acute Myocardial Infarction"[Title/Abstract] OR "Non-ST-Elevation Myocardial Infarction"[Title/Abstract] OR "ST-Elevation Myocardial Infarction"[Title/Abstract] OR "heart infarction"[Title/Abstract]

Risk Factors:

"Risk Factors"[MeSH Terms] OR "Risk Factors"[Title/Abstract] OR "predictors"[Title/Abstract] OR "determinants"[Title/Abstract]

Specific Risk Factors:

"Hypertension"[MeSH Terms] OR "hypertension"[Title/Abstract] OR "high blood pressure"[Title/Abstract]

"Diabetes Mellitus"[MeSH Terms] OR "diabetes"[Title/Abstract] OR "diabetic"[Title/Abstract]

"Coronary Artery Disease"[MeSH Terms] OR "coronary artery disease"[Title/Abstract] OR "coronary heart disease"[Title/Abstract] OR "CAD"[Title/Abstract]

"Coronary Artery Lesions"[Title/Abstract] OR "Coronary Stenosis"[Title/Abstract] OR "Coronary Thrombosis"[Title/Abstract]

"Cystatin C"[Title/Abstract] OR "CysC"[Title/Abstract]

"B-type Natriuretic Peptide"[MeSH Terms] OR "BNP"[Title/Abstract] OR "brain natriuretic peptide"[Title/Abstract]

"Creatine Kinase-MB"[Title/Abstract] OR "CK-MB"[Title/Abstract]

Combination of Risk Factors with Outcomes:

("Hypertension" OR "Diabetes Mellitus" OR "Coronary Artery Disease" OR "Cystatin C" OR "B-type Natriuretic Peptide" OR "Creatine Kinase-MB") AND ("Left Ventricular Remodeling" OR "Cardiac Remodeling") AND ("Myocardial Infarction" OR "Heart Attack")

("Left Ventricular Remodeling"[MeSH Terms] OR "left ventricular remodeling"[Title/Abstract] OR "cardiac remodeling"[Title/Abstract])

AND ("Myocardial Infarction"[MeSH Terms] OR "Myocardial Infarction"[Title/Abstract] OR "Heart Attack"[Title/Abstract])

AND ("Risk Factors"[MeSH Terms] OR "Risk Factors"[Title/Abstract])

AND ("Hypertension" OR "Diabetes Mellitus" OR "Coronary Artery Disease" OR "Cystatin C" OR "B-type Natriuretic Peptide" OR "Creatine Kinase-MB")
